# Comprehensive analysis of key m5C modification-related genes in type 2 diabetes

**DOI:** 10.3389/fgene.2022.1015879

**Published:** 2022-10-06

**Authors:** Yaxian Song, Yan Jiang, Li Shi, Chen He, Wenhua Zhang, Zhao Xu, Mengshi Yang, Yushan Xu

**Affiliations:** ^1^ Department of Endocrinology, The First Affiliated Hospital of Kunming Medical University, Kunming, China; ^2^ Department of Geriatric Medicine, The First Affiliated Hospital of Kunming Medical University, Kunming, China

**Keywords:** bioinformatics, 5-methylcytosine, type 2 diabetes, differentially expressed gene, immune microenvironment, pyroptosis

## Abstract

**Background:** 5-methylcytosine (m5C) RNA methylation plays a significant role in several human diseases. However, the functional role of m5C in type 2 diabetes (T2D) remains unclear.

**Methods:** The merged gene expression profiles from two Gene Expression Omnibus (GEO) datasets were used to identify m5C-related genes and T2D-related differentially expressed genes (DEGs). Least-absolute shrinkage and selection operator (LASSO) regression analysis was performed to identify optimal predictors of T2D. After LASSO regression, we constructed a diagnostic model and validated its accuracy. Gene ontology (GO) and Kyoto Encyclopedia of Genes and Genomes (KEGG) analyses were conducted to confirm the biological functions of DEGs. Gene Set Enrichment Analysis (GSEA) was used to determine the functional enrichment of molecular subtypes. Weighted gene co-expression network analysis (WGCNA) was used to select the module that correlated with the most pyroptosis-related genes. Protein-protein interaction (PPI) network was established using the STRING database, and hub genes were identified using Cytoscape software. The competitive endogenous RNA (ceRNA) interaction network of the hub genes was obtained. The CIBERSORT algorithm was applied to analyze the interactions between hub gene expression and immune infiltration.

**Results:** m5C-related genes were significantly differentially expressed in T2D and correlated with most T2D-related DEGs. LASSO regression showed that *ZBTB4* could be a predictive gene for T2D. GO, KEGG, and GSEA indicated that the enriched modules and pathways were closely related to metabolism-related biological processes and cell death. The top five genes were identified as hub genes in the PPI network. In addition, a ceRNA interaction network of hub genes was obtained. Moreover, the expression levels of the hub genes were significantly correlated with the abundance of various immune cells.

**Conclusion:** Our findings may provide insights into the molecular mechanisms underlying T2D based on its pathophysiology and suggest potential biomarkers and therapeutic targets for T2D.

## Introduction

Diabetes mellitus is a major health problem worldwide, causing life-threatening and disabling complications and lowering life expectancy (Heald et al., 2020). Type 2 diabetes (T2D) accounts for approximately 90% of all diabetes cases worldwide. The 10th edition of the International Diabetes Federation Diabetes Atlas revealed that the prevalence of diabetes worldwide reached 10.5% in 2021 and would rise to 12.2% by 2045 (Sun et al., 2022). In China, the number of T2D patients has been increasing annually, and the disease has become an important public health issue (Li Y. et al., 2020). T2D is a heterogeneous complex disorder. Furthermore, scientific understanding of T2D has resulted in the development of a wider selection of medications. Nevertheless, there is an urgent need to explore the pathophysiology and new treatments to improve early prevention and clinical management of T2D and its complications.

Increasing attention has been given to the role of epigenetic alterations in metabolic diseases. It has been established that epigenetic alterations in certain human tissues contribute to the development of several metabolic disorders, including T2D, and can be a response to the disease (Ling and Rönn, 2019). In addition, epigenetic signatures can be used to diagnose cancer and neurological diseases (Oussalah et al., 2018; Nikolac Perkovic et al., 2021). Recently, many studies have shown that post-transcriptional RNA modifications play an essential role in obesity, diabetes, cardiovascular diseases, cancer, neurological diseases, and other human diseases (Jonkhout et al., 2017; Barbieri and Kouzarides, 2020; Chatterjee et al., 2021). Eukaryotic 5-methylcytosine (m5C) has been well documented as an important form of RNA modification in all RNA species, including mRNAs, rRNAs, tRNAs, and a number of non-coding RNAs (Bohnsack et al., 2019). M5C RNA methylation regulates RNA metabolism by modulating the binding of the writer (methyltransferases), eraser (dimethyltransferases), and reader proteins (Trixl and Lusser, 2019). The m5C modification plays a vital role in RNA translation, transcription, processing, stability, and splicing (Yang et al., 2019; Nombela et al., 2021). Dysregulation and disorder of m5C modification have been associated with multiple human diseases, suggesting that aberrant m5C methylation is correlated with human health (García-Vílchez et al., 2019; Xue et al., 2020). However, the functional role of m5C in T2D remains unclear.

Regulators of m5C modification have attracted attention as potential diagnostic biomarkers or therapeutic tools for T2D (Yanas and Liu., 2019; Chen et al., 2022). In the current study, we explored the possible role of m5C methylation patterns in T2D using bioinformatic analysis. The m5C-related genes and T2D-related differentially expressed genes (DEGs) were analyzed using the merged gene expression profiles from two Gene Expression Omnibus (GEO) datasets. We determined the potential signaling pathways of the molecular subtypes. An m5C-related gene diagnostic model for predicting the risk of T2D was constructed, and the diagnostic value of the model was assessed. The study also explored the association between m5C-related genes and genes in the pyroptosis module. Based on this, we selected the top five hub genes based on the protein-protein interaction network. Moreover, we constructed a competitive endogenous RNA (ceRNA) regulatory network related to hub m5C-related genes in T2D. Finally, we assessed the effect of hub m5C-related genes on immune infiltration in T2D using CIBERSORT.

## Materials and methods

### GEO datasets processing

Microarray data and sample information from two gene expression profile datasets (GSE29221 and GSE182120) were downloaded from the GEO (Jain et al., 2013; Gabriel et al., 2021). GSE29221 (Jain et al., 2013) was based on the GPL6947 platform Illumina HumanHT-12 V3.0 expression BeadChip (*Homo sapiens*). It also contained data from 24 human skeletal muscle samples, of which 12 were from T2D patients and 12 were from patients without diabetes. For the GSE29221 dataset, 48803 probe IDs were converted to 25159 gene symbols according to platform annotation files. GSE182120 (Gabriel et al., 2021) was obtained using the GPL17586 platform [HTA-2_0] Affymetrix Human Transcriptome Array 2.0. It also contained data from 49 human skeletal muscle samples, of which 25 were from T2D patients and 24 were from patients without diabetes. For the GSE182120 dataset, 70523 probe IDs were converted to 30905 gene symbols according to platform annotation files. All samples were extracted, the GSE182120 data were log-normalized, and the GSE29221 data were normalized using Z-score transformation. The level of gene expression changes in different samples and conditions was detected based on the gene expression data normalized by Z-transformation (Cheadle et al., 2003). The batch effect was eliminated using the sva package, which contained functions for adjusting known batch effects and latent variables in prediction problems (Leek et al., 2012). Boxplots to visualize expression distribution were generated using the R package ggplot2 (Wickham, 2011). After merging the data, the dataset contained 16414 genes from 37 T2D and 36 normal samples.

### DEG analysis and correlation analysis of m5C

To identify the differences of m5C in human skeletal muscle tissues, the samples were assigned to T2D and control groups. First, we obtained a list of m5C-related genes by reviewing previous literature (Du et al., 2020; Zhang et al., 2021) regarding the m5C RNA, including 11 m5C writer genes (*NSUN1*, *NSUN2*, *NSUN3*, *NSUN4*, *NSUN5*, *NSUN6*, *NSUN7*, *DNMT1*, *DNMT2*, *DNMT3A*, and *DNMT3B*), 16 m5C reader genes (*ALYREF*, *YBX1*, *MBD1*, *MBD2*, *MBD3*, *MBD4*, *MECP2*, *NEIL1*, *NTHL1*, *SMUG1*, *TDG*, *UHRF1*, *UHRF2*, *UNG*, *ZBTB33*, and *ZBTB4*), and three m5C eraser genes (*TET1*, *TET2*, and *TET3*). A total of 26 m5C-related genes overlapped with genes from the merged dataset. Heatmaps depicting m5C-related genes and boxplots were plotted using the pheatmap (Kolde, 2019), the ggplot2 (Wickham, 2011) and ggpubr (Kassambara, 2020) R packages, respectively. We used the Wilcoxon signed-rank test to confirm the expression changes between the control and T2D groups (*p* < 0.05).

To examine the relationships between m5C- and T2D-related genes, we first conducted DEG analysis using the limma package (Ritchie et al., 2015) of the R program. T2D-related DEGs were defined as upregulated genes with fold changes (FC) above 1.2 or downregulated genes with FC lower than 0.83, with *p* < 0.05 (Zhang et al., 2019). The expression pattern of T2D-related DEGs was established as a volcano plot using the ggplot2 package. Correlations between m5C-related gene expression and T2D-related DEG expression were determined using Pearson’s correlation test. Pearson correlation coefficient heatmaps were visualized using the “corrplot” package (Weiet al., 2021). Pearson correlation coefficients were calculated among m5C-related genes in all samples and were displayed using Cytoscape software (Shannon et al., 2003).

### Construction of the m5C-related gene diagnostic model

Because of the important influence of the m5C modification process, healthy and T2D groups may have different m5C modification states, so it was viable to construct a diagnostic model dependent on m5C-related genes.

We performed least-absolute shrinkage and selection operator (LASSO) regression using the “glmnet” package (Friedman et al., 2010) in R to determine whether the occurrence of diabetes was the dependent variable (control = 0, T2D = 1). Only genes with nonzero regression coefficients were considered.

To check the multi-factor influence of high-weight genes in the diagnostic model, a new logistic multi-factor regression model was constructed for m5C-related genes screened from the previous model using the glmnet R package, and the prediction score was visualized between the two groups using the ggpubr R package. In addition, we constructed the receiving operating characteristic (ROC) curves and calculated the areas under the ROC curve (AUC) to assess the model predictive performance (R package pROC) (Robin et al., 2011).

### Unsupervised clustering of samples

Owing to the prevalence of heterogeneity between patients, unsupervised clustering of samples based on 26 m5C-related genes can resolve this heterogeneity and reclassify the samples. Unsupervised consensus clustering of samples was performed based on an aggregation hierarchical clustering algorithm using the ConsensusClusterPlus package in R (Leek et al., 2012). The optimal number of clusters was determined by calculation. To validate unsupervised clustering results, principal component analysis (PCA) was performed and visualized using the ggfortify R package (Horikoshi and Yuan, 2022). The ggplot2 and ggpubr packages were used to draw boxplots showing the differential gene expression between molecular subtypes based on sample clustering. To verify the validity of molecular subtypes, statistical analyses were performed using the Wilcoxon signed-rank test, with *p* < 0.05.

### Functional annotation of DEGs

DEGs between molecular subtypes were recognized as FC > 1.2 for upregulated genes or FC < 0.83 for downregulated genes, with *p* < 0.05. These DEGs were used for the enrichment analysis.

Gene Ontology (GO) enrichment analysis, including biological process (BP), molecular function (MF), and cellular component (CC), is a commonly used method for functional annotation of genes (Ashburner et al., 2000). Kyoto Encyclopedia of Genes and Genomes (KEGG) is a database resource providing genomic and molecular information (Kanehisa and Goto, 2000). KEGG pathway analysis is widely used in bioinformatics to annotate and enrich the pathways. GO and KEGG pathway analyses were performed on the DEGs between molecular subtypes using the clusterProfiler R package (Yu et al., 2012; Wu et al., 2021), with *p* < 0.05 as a significance threshold. The results of the enrichment analysis were visualized using bubble plots.

### Gene set enrichment analysis (GSEA) and gene set variation analysis (GSVA)

GSEA is a computational software that assesses whether a prior-defined set of genes shows statistical differences between two biological states and is used to estimate biological processes and pathways (Subramanian et al., 2005). To study the differences in biological processes between the two molecular typing samples, enrichment analysis and visualization were conducted using the GSEA function in the R package clusterProfiler (Yu et al., 2012; Wu et al., 2021). Differences were considered statistically significant when the corrected *p*-value was less than 0.05. GSVA is a non-parametric unsupervised method that estimates variations in GSEA by transforming a matrix of gene expression across samples into a matrix of gene set enrichment scores across the same samples (Hänzelmann et al., 2013). GSEA was performed using the GSVA R package with gene sets (c5. go.v7.4. entrez_input) obtained from MSigDB database (Liberzon et al., 2015). Differential analysis was carried out using the limma package in R (Ritchie et al., 2015). Differential pathways were defined as absolute values of log_2_ FC > 0.02 and *p*-value < 0.05 (Li et al., 2021). Heatmaps were drawn using the pheatmap R package.

### Correlations of the m5C-related DEGs

To examine the relevance of m5C-related DEGs between the two molecular subtypes, Wilcoxon rank sum tests were performed with *p* < 0.05 (Tan et al., 2002), and boxplots were created using the ggpubr package. Correlations among the expression of m5C-related DEGs were calculated using Pearson’s correlation coefficient. When analyzing correlations, Spearman’s correlation coefficient was considered significant if the *p*-value was less than 0.05, and the absolute value of the correlation coefficient larger than 0.8 was considered a strong correlation. Scatter plots and fitting curves were constructed in R using the ggplot2 package. Histograms were made in R using the ggExtra package (Attali and Baker, 2022).

### Weighted gene co-expression network analysis (WGCNA)

WGCNA is a method for clustering genes into modules based on correlations among gene expression patterns. Co-expression networks were constructed using the WGCNA package (Langfelder and Horvath, 2008) (R square value = 0.8, an appropriate soft threshold to identify clusters). The gray modules represent genes that had not been classified and, therefore, these were deleted. Because of the important effects of pyroptosis on diabetes (Lu et al., 2021), we retrieved pyroptosis-related genes from the GeneCards database (Stelzer et al., 2016), searching for the keyword criteria “pyroptosis”. Module analysis and mining were performed with the characteristics of pyroptosis-related genes to obtain modules significantly related to pyroptosis for further analysis.

### Protein-protein interaction (PPI) establishment and identification of hub genes

As pyroptosis and m5C have important effects on diabetes (Liu et al., 2021; Lu et al., 2021), we determined if any genes of the pyroptosis module overlapped with m5C-related genes. A PPI network was constructed based on overlapping genes using the STRING database (von Mering et al., 2003). The PPI pairs were identified using the confidence threshold (default value of 0.4). The degree of each node was calculated using the CytoHubba plugin for Cytoscape (Chin et al., 2014). Hub genes were defined as nodes within the top five-degree values in a network. These nodes have a high level of connection with other nodes and thus may play a crucial role in regulating the entire biological process.

### Construction of ceRNA regulatory network

A lncRNA-miRNA-mRNA network was constructed according to the “competing of ceRNA” hypothesis.

Correlations between the hub gene expression and lncRNA expression profiles were assessed. The correlation coefficients and significance values (*p*) were evaluated using the Hmisc package in R (Harrell and Dupont, 2022). The interacting pairs that had absolute value of r larger than 0.3 and *p* ˂ 0.05 were assumed to be significantly correlated. A list of experimentally validated human lncRNA-miRNA and miRNA-mRNA interaction pairs was downloaded from the mirTarBase database (Huang et al., 2020). After analyzing the overlap of the significantly correlated interaction pairs with those of the list, the lncRNA-miRNA-mRNA ceRNA network was generated using the Cytoscape software.

### Correlation analysis of hub genes and immune infiltration

Immune cells are essential components of the immune microenvironment and play fundamental roles in the development of diseases. Immune cell infiltration in tissues plays a significant and instructive role in disease development and prognosis prediction. To further explore the relationship between hub genes and immune cell infiltration, we dissected the proportion of immune cells in each sample using CIBERSORT (Chen et al., 2018) and the LM22 signature matrix. Boxplots were constructed using the ggpubr package. To investigate the correlations between hub gene expression and immune infiltration levels, we used the ggpubr package to generate boxplots based on 22 immune cell subtypes. Statistical comparisons were performed using the Wilcoxon signed-rank test, with *p* < 0.05.

### Statistical analyses

All data calculations and statistical analyses were performed using R (version 4.0.2). To compare the two groups of continuous variables, the statistical significance of the normally distributed variables was estimated using the independent *t*-test. To compare the two groups of non-normally distributed independent variables, the difference was analyzed using the Wilcoxon rank sum test for non-normally distributed independent variables. ROC curves were plotted, and AUC values were calculated using the R package pROC. All statistical tests were 2-sided, and *p* < 0.05.

**FIGURE 1:**
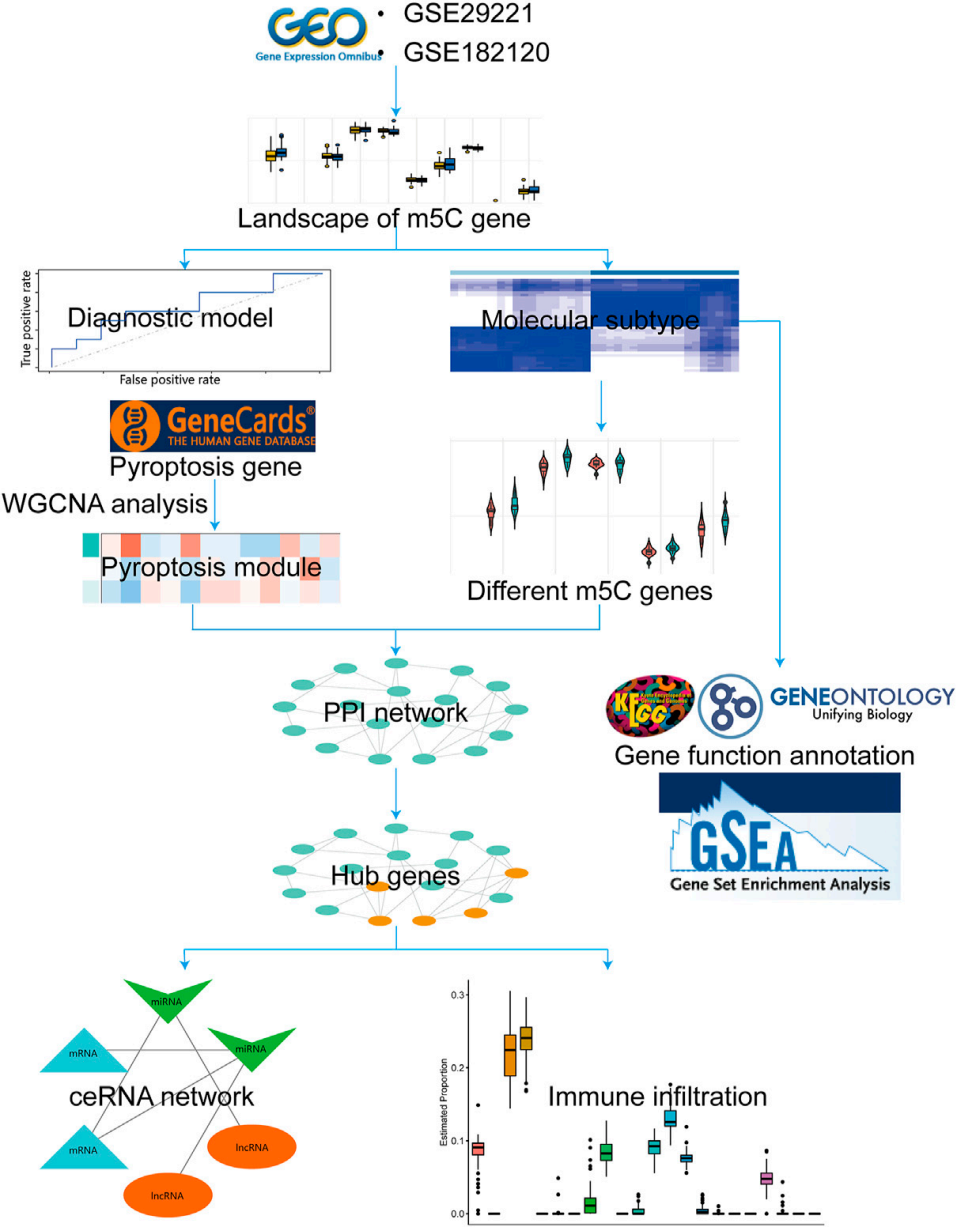
Workflow for the whole study.

Overall study design and methodologies were summarized as a workflow (Figure 1).

## Results

### Identification of m5C-related genes

**FIGURE 2:**
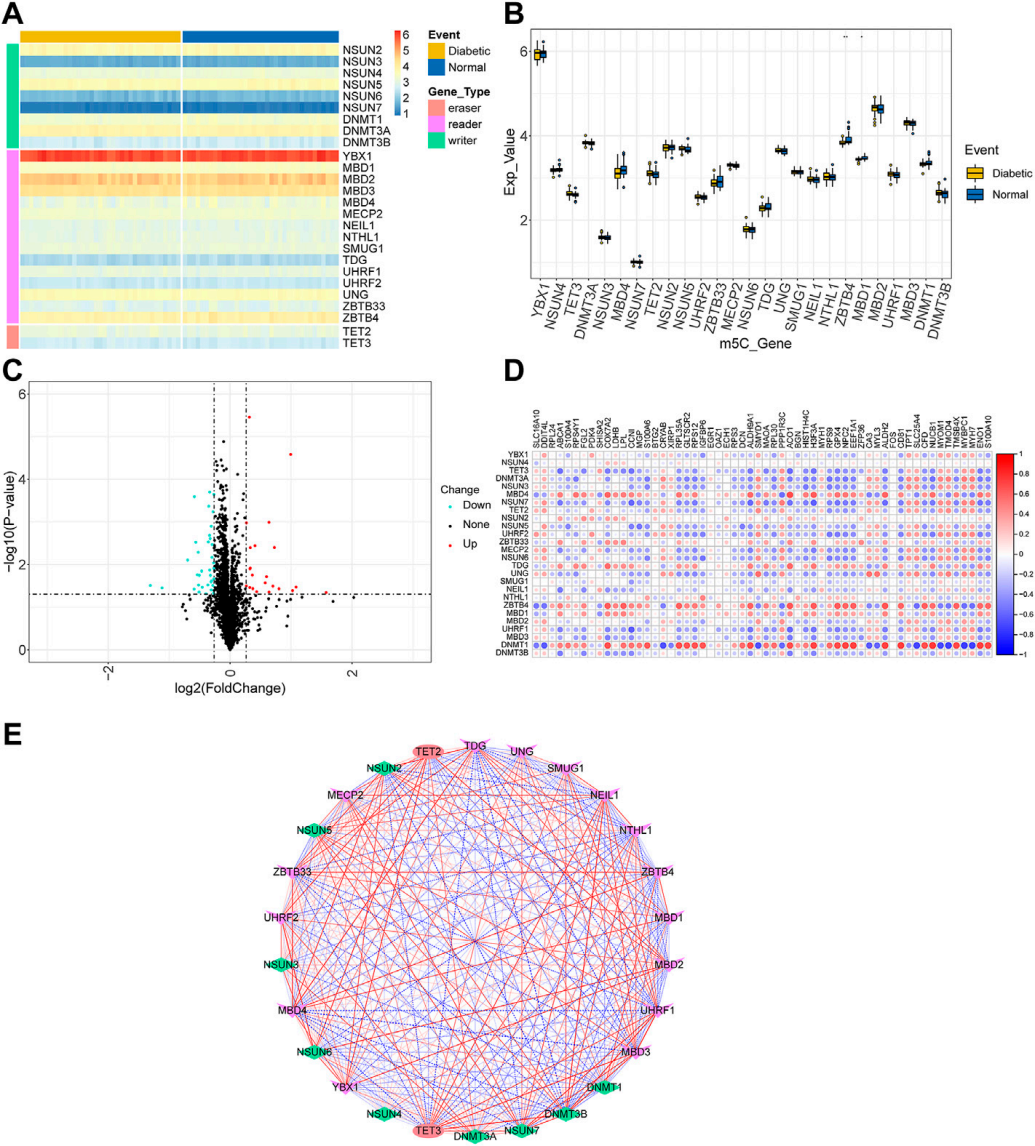
Landscape of m5C-related genes. **(A)** Heatmap of the m5C-related genes. **(B)** Boxplot of m5C-related genes. Ns indicates not significant (*p* > 0.05), and *p < 0.05, **p <0.01, ***p <0.001, ****p < 0.0001 represent significant *p*-values. **(C)** Volcano plot of T2D-related differentially expressed genes. **(D)** Correlations among m5C-related genes and T2D-related differentially expressed genes. **(E)** Network diagram of the correlation interaction between m5C-related genes.

First, m5C-related gene expression maps were established. The boxplots (Supplementary Figure S1) showed that the expression distribution of all samples were consistent after batch correction. This assured the accuracy of downstream analysis. Heatmaps and boxplots were drawn according to the expression matrix of the samples to show the difference in m5C-related gene expression between the control and T2D groups (Figures 2A,B). The m5C-related genes between the two groups were identified using a two-group comparison (Supplementary Table S2). The *TET* gene family was highly expressed in the T2D group, while the *ZBTB* gene family was highly expressed in the control group.

Then, to explore the relationship between m5C-related genes and T2D-related DEGs, a total of 58 T2D-related DEGs were screened (Supplementary Table S1) and visualized using a volcano plot (Figure 2C). Figure 2D depicts the Pearson’s correlation coefficients between m5C-related genes and T2D-related DEGs. M5C-related genes were significantly correlated with most T2D-related DEGs such as *DNMT1-NUCB1* (r = 0.758, *p* = 8.75e-15) and *DNMT1-MYBPC1* (r = -0.741, *p* = 6.29e-14). These results suggest that the m5C-related genes are associated with diabetes. Using Pearson’s correlation, we found notable positive and negative correlation in the expression among m5C-related genes (Figure 2E), including UHRF1-TET3 (r = 0.855, *p* = 5.82e-22) and DNMT3B-TDG (r = -0.751, *p* = 2.02e-14). In conclusion, these results suggest that m5C-related genes may regulate T2D-related DEGs through positive or negative regulatory interactions.

### Construction of diagnostic model based on m5C-related genes

**FIGURE 3:**
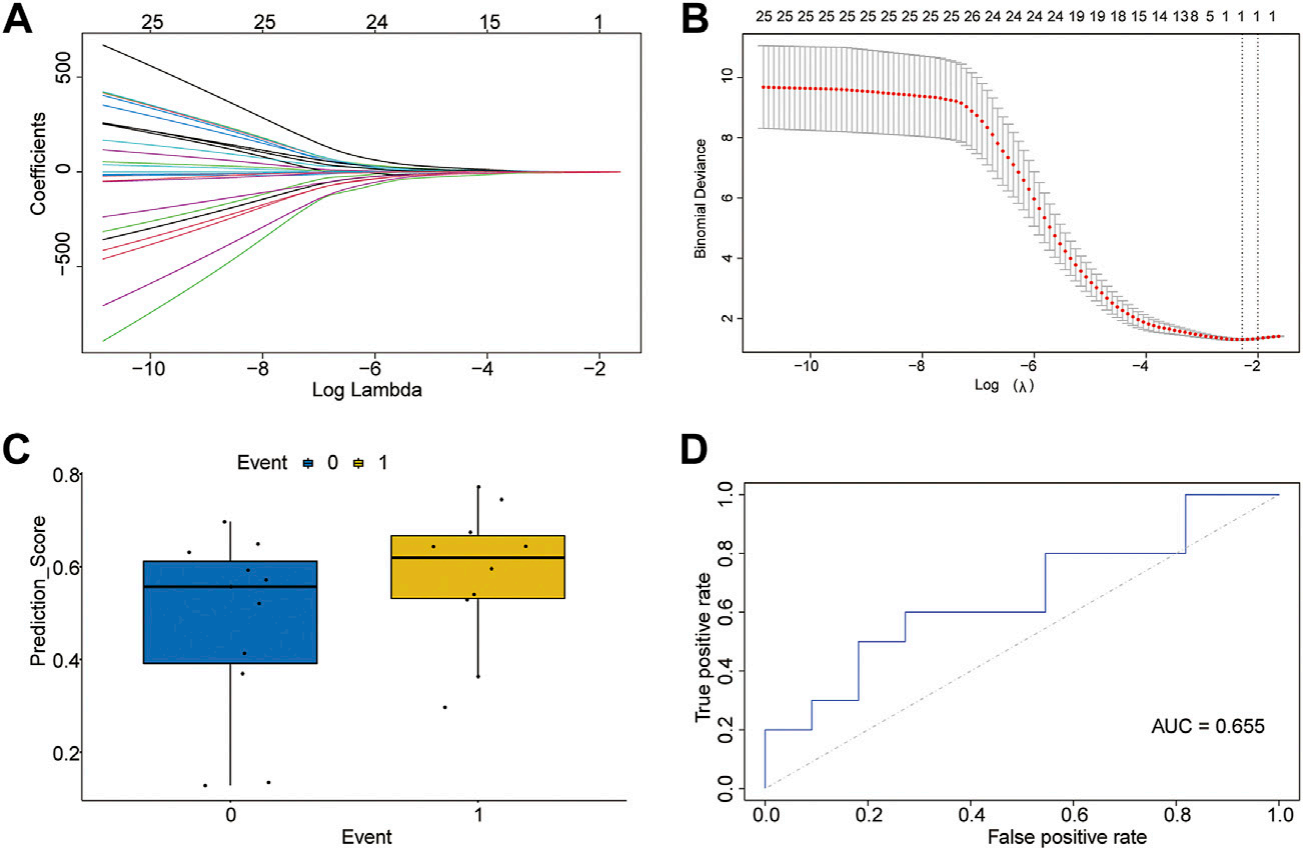
Diagnostic model of m5C-related genes. **(A)** LASSO regression curve. **(B)** Curve for selection of lambda value. **(C)** Predicted effect of the LASSO regression model (T2D = 1, control = 0). **(D)** Receiver operating characteristic curve of the logistic model for diagnosis of T2D.

LASSO regression was performed on 26 m5C-related genes. The lambda value with minimal average deviance was determined as the optimal lambda (0.1,019,701) by cross-validation (Figures 3A,B). We found that only the *ZBTB4* gene was ultimately retained. Then, logistic regression was used to develop a diagnostic model containing the *ZBTB4* gene and estimate regression coefficients. The prediction scores of the diagnostic model differed between the control and T2D groups (Figure 3C). A multivariate logistic regression model was constructed to validate the accuracy of the diagnostic model. The predictive model showed acceptable diagnostic performance with an AUC value of 0.655 (Figure 3D).

### Unsupervised clustering of samples

**FIGURE 4:**
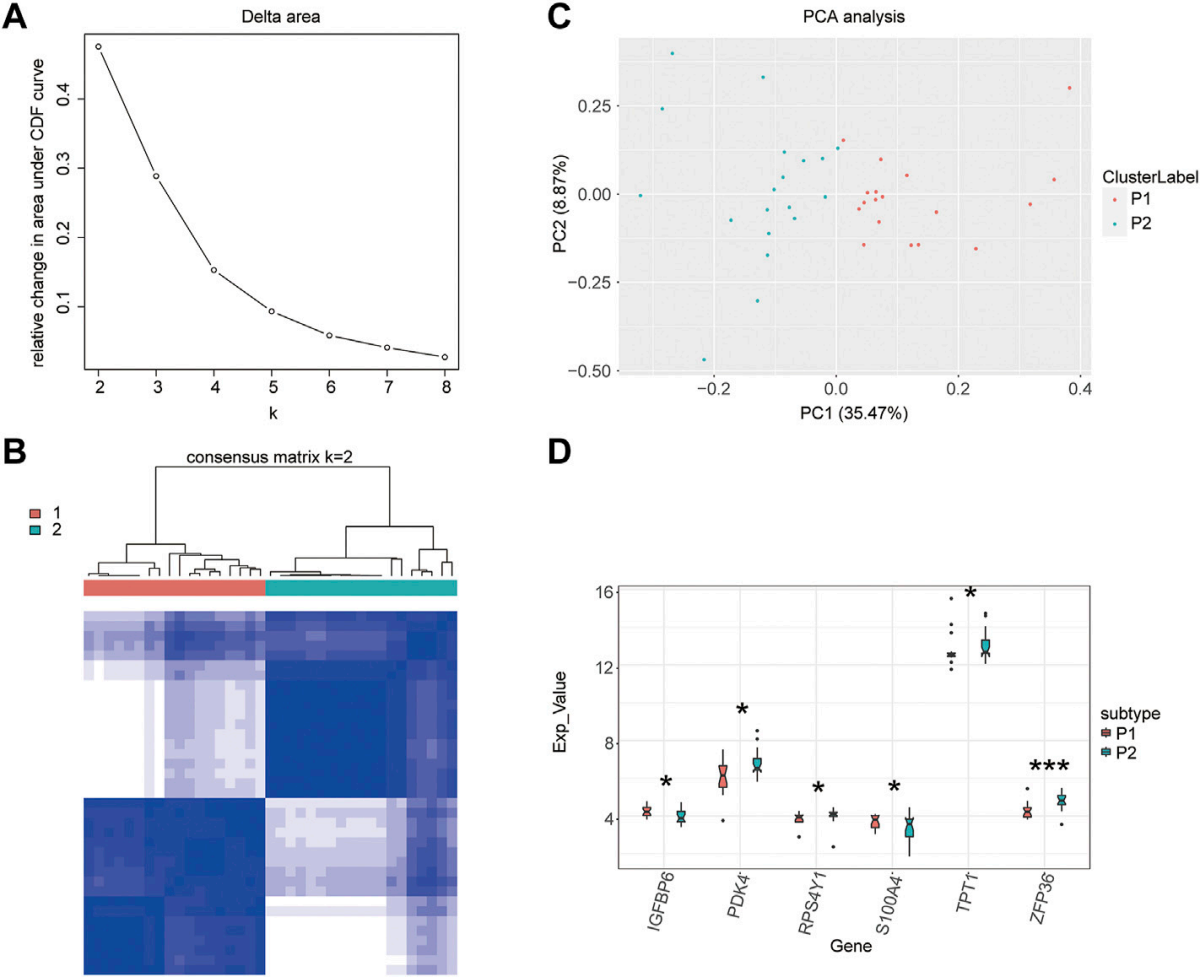
Unsupervised clustering of samples. **(A)** Selection of the optimal clustering number. **(B)** Clustering heatmap. **(C)** Principal component analysis. **(D)** Boxplot of the clustering.

An unsupervised clustering of the 26 m5C-related genes for the diabetic samples was performed. Two clusters had the best clustering effect (Figure 4A); therefore, we used K = 2 as the optimal number of clusters to perform unsupervised clustering and clustered the diabetic samples into two categories. Then, the clustering effect was visualized using clustering heatmaps (Figure 4B). The principal component analysis conducted to verify the unsupervised clustering results showed that m5C-related genes could effectively separate the two molecular subtypes (Figure 4C), demonstrating the accuracy of clustering results. Finally, the Wilcoxon rank-sum test was used to examine the expression levels of T2D-related DEGs in the two molecular subtypes. *IGFBP6*, *PDK4*, *RPS4Y1*, *S100A4*, *TPT1*, and *ZFP63* genes showed significant differences in their expression levels between the two molecular subtypes (Figure 4D). These results indicated that these genes were also related to their typing, which also reflected the validity and accuracy of the clustering results.

### GO and KEGG pathway enrichment analyses of DEGs

**FIGURE 5:**
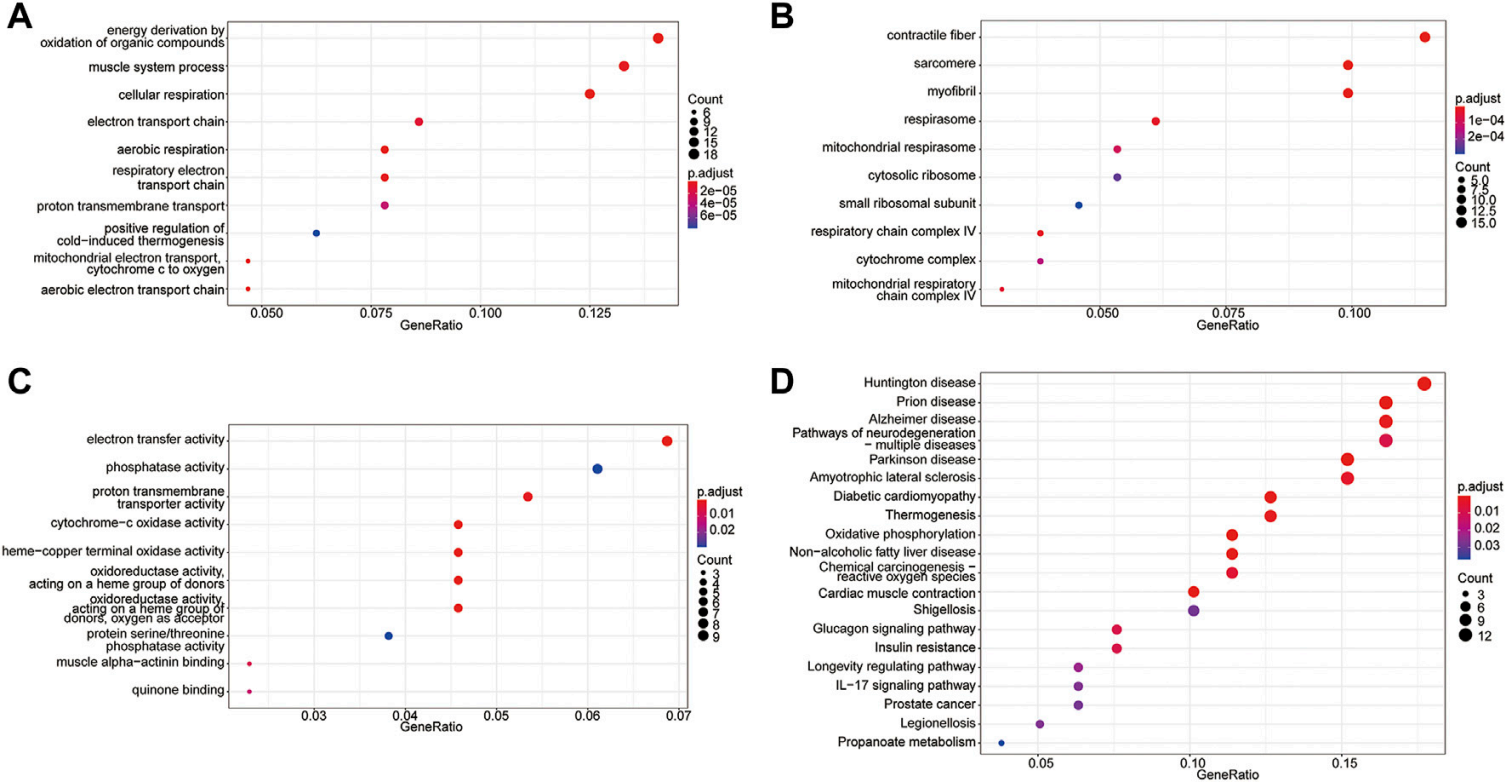
GO terms and KEGG pathway enrichment analyses of DEGs. **(A)** The top 10 significantly enriched biological processes. **(B)** The top 10 significantly enriched cellular components. **(C)** The top 10 significantly enriched molecular functions. **(D)** KEGG pathway analysis.

To probe the biological functions of the molecular subtypes, all DEGs between the two molecular subtypes were subjected to GO and KEGG enrichment analyses. A total of 120 enriched GO terms were observed in GO enrichment analyses. The top five most significant terms were cellular respiration, energy derivation by the oxidation of organic compounds, contractile fiber, sarcomere, and aerobic respiration. These results involved multiple biological processes related to energy metabolism and further confirmed the accuracy of the identified gene set (Figure 5A-C, Table 1). KEGG pathway analysis revealed that 24 pathways were enriched, including oxidative phosphorylation, thermogenesis, propanoate metabolism, and carbon metabolism pathways. These results can also complement the GO enrichment results. This further demonstrated that these genes were associated with metabolism-related biological processes or functions (Figure 5D, Table 2).

**TABLE 1:** GO enrichment analyses. (Only the top 15 terms are shown).

Ontology	Description	Adjusted *p*-value
BP	cellular respiration	3.97E-10
BP	energy derivation by oxidation of organic compounds	6.78E-10
CC	contractile fiber	1.27E-08
CC	sarcomere	1.58E-07
BP	aerobic respiration	2.72E-07
CC	myofibril	3.50E-07
BP	mitochondrial electron transport, cytochrome c to oxygen	2.13E-06
BP	aerobic electron transport chain	2.13E-06
BP	respiratory electron transport chain	2.87E-06
MF	cytochrome-c oxidase activity	3.41E-06
MF	heme-copper terminal oxidase activity	3.41E-06
MF	oxidoreductase activity, acting on a heme group of donors	3.41E-06
MF	oxidoreductase activity, acting on a heme group of donors, oxygen as acceptor	3.41E-06
BP	muscle system process	4.08E-06
BP	electron transport chain	1.11E-05

**TABLE 2:** KEGG enrichment analyses.

ID	Description	*p*-value
hsa05016	Huntington disease	1.14E-06
hsa04260	Cardiac muscle contraction	1.72E-06
hsa05020	Prion disease	1.84E-06
hsa00190	Oxidative phosphorylation	5.24E-06
hsa05012	Parkinson disease	8.37E-06
hsa04932	Non-alcoholic fatty liver disease	1.71E-05
hsa05415	Diabetic cardiomyopathy	2.39E-05
hsa05010	Alzheimer disease	7.18E-05
hsa04714	Thermogenesis	7.44E-05
hsa05014	Amyotrophic lateral sclerosis	0.000180368
hsa05208	Chemical carcinogenesis - reactive oxygen species	0.000286287
hsa04922	Glucagon signaling pathway	0.000570642
hsa05022	Pathways of neurodegeneration - multiple diseases	0.000596652
hsa04931	Insulin resistance	0.000599597
hsa04211	Longevity regulating pathway	0.001660804
hsa04657	IL-17 signaling pathway	0.002115276
hsa05134	Legionellosis	0.002193253
hsa05215	Prostate cancer	0.002428137
hsa05131	Shigellosis	0.002597656
hsa00640	Propanoate metabolism	0.003557727
hsa03010	Ribosome	0.004196813
hsa04920	Adipocytokine signaling pathway	0.0043975
hsa05417	Lipid and atherosclerosis	0.004694059
hsa01200	Carbon metabolism	0.005052249

### GSEA and GSVA

**FIGURE 6:**
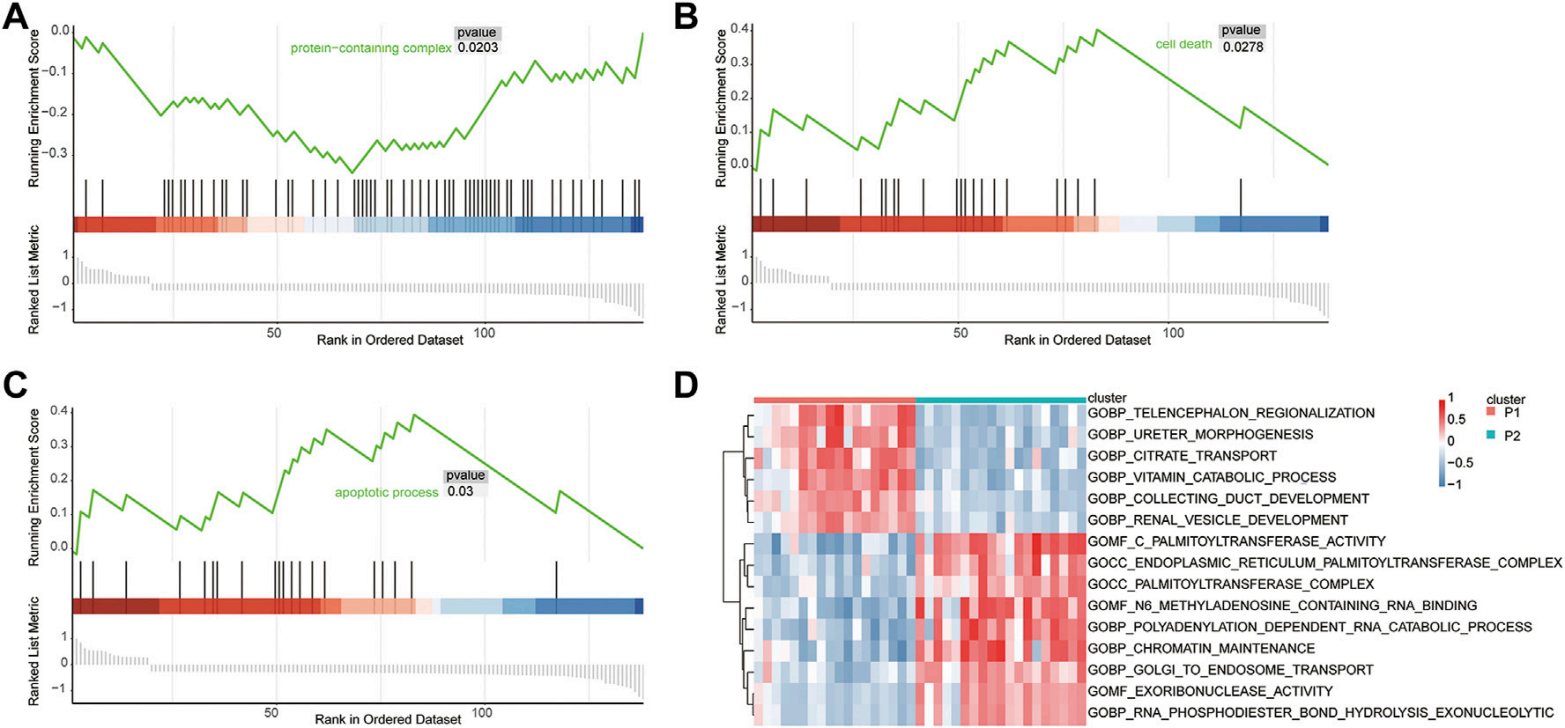
GSEA and GSVA. Gene set enrichment analysis demonstrating the protein-containing complex **(A)**, cell death **(B)**, and apoptotic process **(C)** signaling pathways enrichment in T2D. **(D)** GSVA analysis of differential pathway.

GSEA was used to identify the functional enrichment of the two molecular subtype. Six significant GO-terms were found to be enriched (Figure 6A-C, Table 3). These include protein-containing complexes, cell death, and apoptosis process. These results validated and complemented our GO and KEGG analysis results. In addition, the GSVA method was used to assess the significance of pathway alterations in diabetic samples. We computed pathway expression scores for each sample and identified 4,091 pathways with significant differences. These included palmitoyltransferase activity, collecting duct development, and telencephalon regionalization (Figure 6D, Table 4).

**TABLE 3:** GSEA.

Description	NES	*p*-value
protein-containing complex	-1.630309425	0.02027027
cell death	1.756117512	0.027777778
apoptotic process	1.72075552	0.03003003
programmed cell death	1.72075552	0.03003003
regulation of cell death	1.669531533	0.031700288
regulation of apoptotic process	1.617003364	0.045714286

**TABLE 4:** GSVA. (Only the top 10 pathways are shown).

Description	logFC	*p*-value	Adjusted *p*-value
GOMF_C_PALMITOYLTRANSFERASE_ACTIVITY	-0.903282932	2.19E-12	2.23E-08
GOBP_COLLECTING_DUCT_DEVELOPMENT	0.583850809	5.18E-12	2.63E-08
GOBP_TELENCEPHALON_REGIONALIZATION	0.723419914	1.46E-11	4.93E-08
GOCC_ENDOPLASMIC_RETICULUM_PALMITOYLTRANSFERASE_COMPLEX	-0.697389902	2.34E-11	5.93E-08
GOMF_EXORIBONUCLEASE_ACTIVITY	-0.584344635	3.55E-11	7.20E-08
GOCC_PALMITOYLTRANSFERASE_COMPLEX	-0.621978615	5.45E-11	9.22E-08
GOBP_RNA_PHOSPHODIESTER_BOND_HYDROLYSIS_EXONUCLEOLYTIC	-0.572339064	1.36E-10	1.97E-07
GOBP_URETER_MORPHOGENESIS	0.699920401	1.86E-10	2.07E-07
GOMF_N6_METHYLADENOSINE_CONTAINING_RNA_BINDING	-0.835486369	2.03E-10	2.07E-07
GOBP_CITRATE_TRANSPORT	0.723749741	2.10E-10	2.07E-07

### Correlations of the m5C-related DEGs expression

**FIGURE 7:**
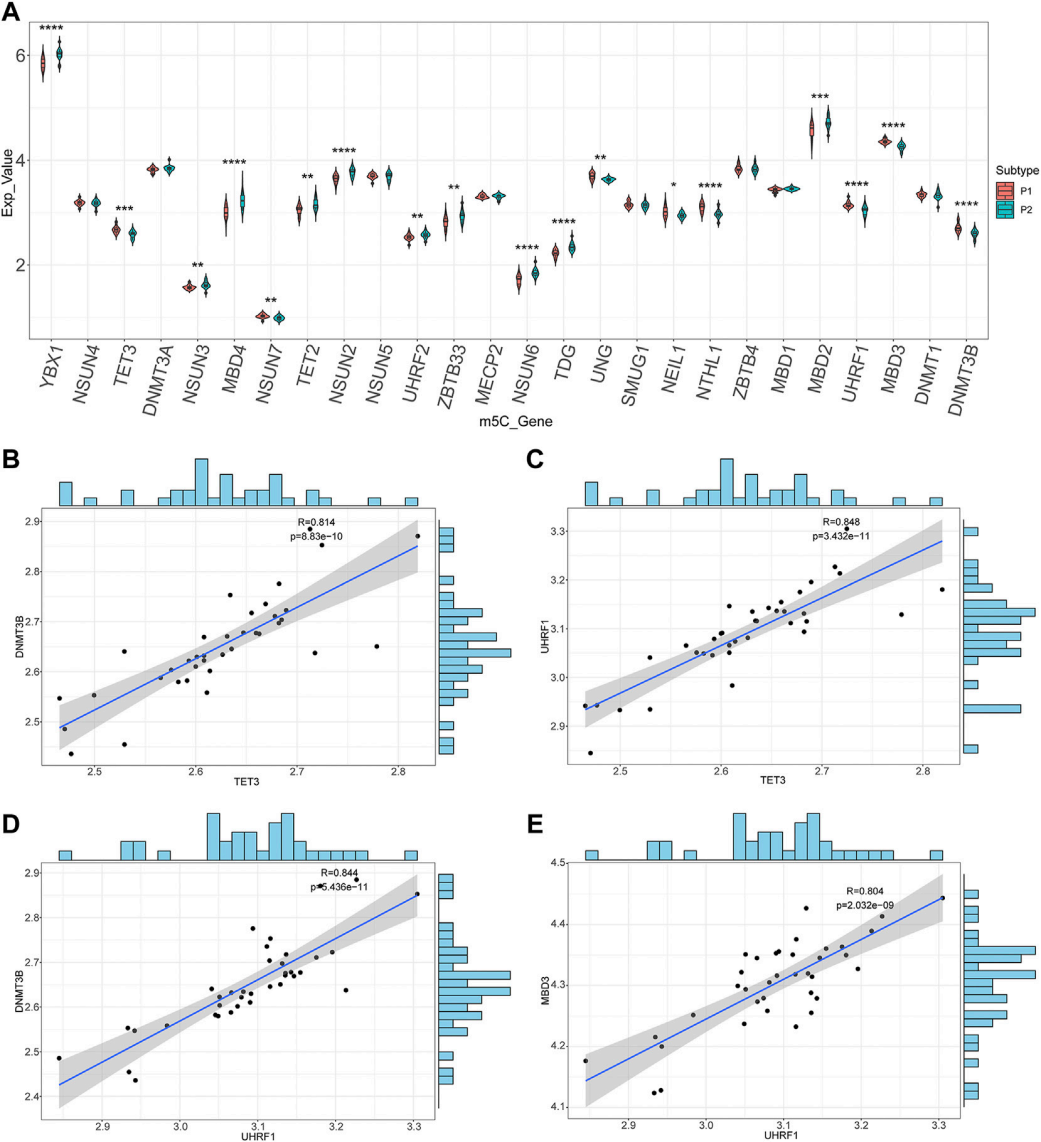
Correlations of m5C-related genes. **(A)** The boxplots of the m5C-related genes between two molecular subtypes. **(B–E)** Scatter plots and the fitting curves of the differentially expressed m5C-related genes.

To further analyze the correlations of m5C-related DEGs expression between the two molecular subtypes, the Wilcoxon rank-sum test was used to identify 18 m5C-related DEGs: *YBX1*, *TET3*, *NSUN3*, *MBD4*, *NSUN7*, *TET2*, *NSUN2*, *UHRF2*, *ZBTB33*, *NSUN6*, *TDG*, *UNG*, *NEIL1*, *NTHL1*, *MBD2*, *UHRF1*, *MBD3*, and *DNMT3B* (Figure 7A). After calculating the correlation coefficients between any two m5C-related DEGs, correlation scatter plots and fitted curves were generated. Four gene pairs met the statistical significance threshold: *TET3*-*DNMT3B* (r = 0.814, *p* = 8.83e-10), *TET3*-*UHRF1* (r = 0.848, *p* = 3.43e-11), *UHRF1*-*DNMT3B* (r = 0.844, *p* = 5.44e-11), and *UHRF1*-*MBD3* (r = 0.804, *p* = 2.03e-09) (Figures 7B–E). Overall, *UHRF1*, *TET3*, and the other genes were significantly positively correlated. This indicated an interaction between the m5C-related genes based on patient typing.

### WGCNA

**FIGURE 8:**
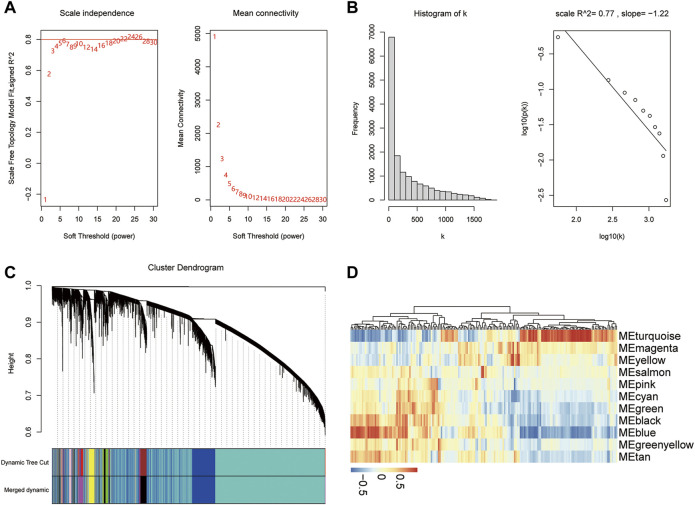
WGCNA. **(A)** Analysis of the scale-free index and the mean connectivity for various soft-threshold powers. **(B)** Checking the scale-free topology when *β* = 6. **(C)** Dendrogram of the pyroptosis-related genes. The color band shows the results obtained from the automatic single-block analysis. **(D)** Heatmap of the correlations between the modules and traits of pyroptosis.

To determine the relationship between diabetes and pyroptosis, the R package WGCNA was used to cluster the samples in this study. First, an appropriate soft threshold was selected using the pickSoftThreshold function and was set at 6 (Figure 8A). In this study, *β* = 6 was chosen to construct the network, at which time the correlation coefficient between log(k) and log (p(k)) was close to 0.8 (Figure 8B). We constructed a hierarchical clustering tree using a dynamic hybrid tree–cut algorithm. Every leaf in the tree corresponded to a gene, and a branch of the tree represented a gene module, which meant that these genes had similar expression data, and a total of 11 modules were generated (Figure 8C). Among the 11 modules, the turquoise module was associated with the most pyroptosis-related genes (|r| > 0.3, *p* < 0.05), indicating that it was more relevant to pyroptosis (Figure 8D). We chose the genes in the turquoise module for further analysis.

### PPI network establishment and identification of hub genes

**FIGURE 9:**
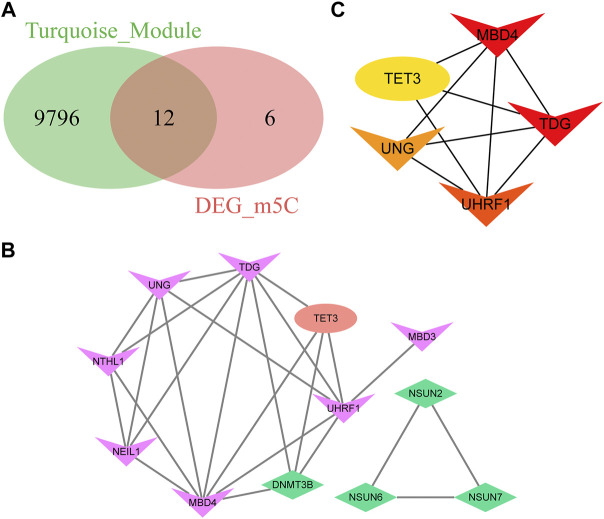
Protein-protein interaction and identification of hub genes. **(A)** Venn diagram of the differentially expressed m5C-related genes in the turquoise module. **(B)** Protein-protein interaction network of the differentially expressed m5C-related genes in the turquoise module. **(C)** CytoHubba plugin analysis of the top five hub genes with the degree algorithm.

Genes that regulate the same biological processes are closely related because of their widespread linkage. To further analyze the differentially expressed m5C-related genes in the pyroptosis module, we first intersected the genes in the turquoise module with m5C-related DEGs and obtained 12 genes, namely, *TET3*, *MBD4*, *NSUN7*, *NSUN2*, *NSUN6*, *TDG*, *UNG*, *NEIL1*, *NTHL1*, *UHRF1*, *MBD3*, and *DNMT3B* (Figure 9A). Next, we set up a PPI network using the STRING database, which included 12 genes and 24 interaction relationships. The average node degree was four in the PPI network, with an enriched *p*-value < 1.0e-16, clustering coefficient of 0.73, density of 0.583, and centralization of 0.375. Subsequently, visualization of the PPI network was completed using Cytoscape software (Figure 9B).

In the network, a few nodes were more closely connected with the remaining nodes; that is, these genes will have more impact on the whole network, emphasizing the significance of the network. Using the plugin Cytohubba in Cytoscape software, the top five genes were defined as hub genes from the PPI network: *MBD4*, *TDG*, *UHRF1*, *UNG*, and *TET3* (Figure 9C).

### Construction of ceRNA network

**FIGURE 10:**
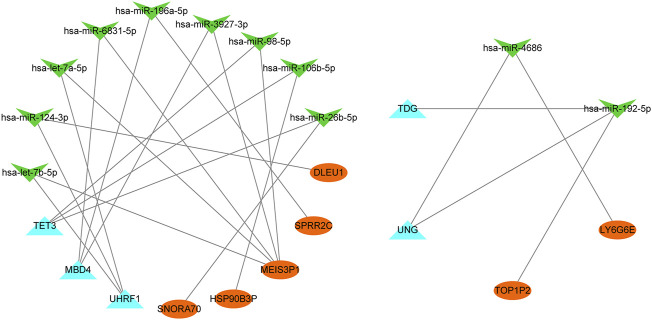
CeRNA network. MiRNAs (green arrow), m5C-related genes (blue triangle), and lncRNAs (orange oval) are represent.

Seven lncRNAs (lncRNA SNORA70, HSP90B3P, MEIS3P1, SPRR2C, DLEU1, lncRNA TOP1P2, and LY6G6E), 11 miRNAs (has-let-7b-5p, has-miR-124-3p, has-let-7a-5p, has-miR-6831-5p, has-miR-196a-5p, has-miR-3927-3p, has-miR-98-5p, has-miR-106b-5p, has-miR-26b-5p, has-miR-4686, and has-miR-192-5p), and five mRNAs (TET3, MBD4, UHRF1, UNG, and TDG) were identified (Figure 10). These results suggest several post-transcriptional regulatory programs for expressing key genes in diabetes.

### Assessment of the immune microenvironment in T2D

**FIGURE 11:**
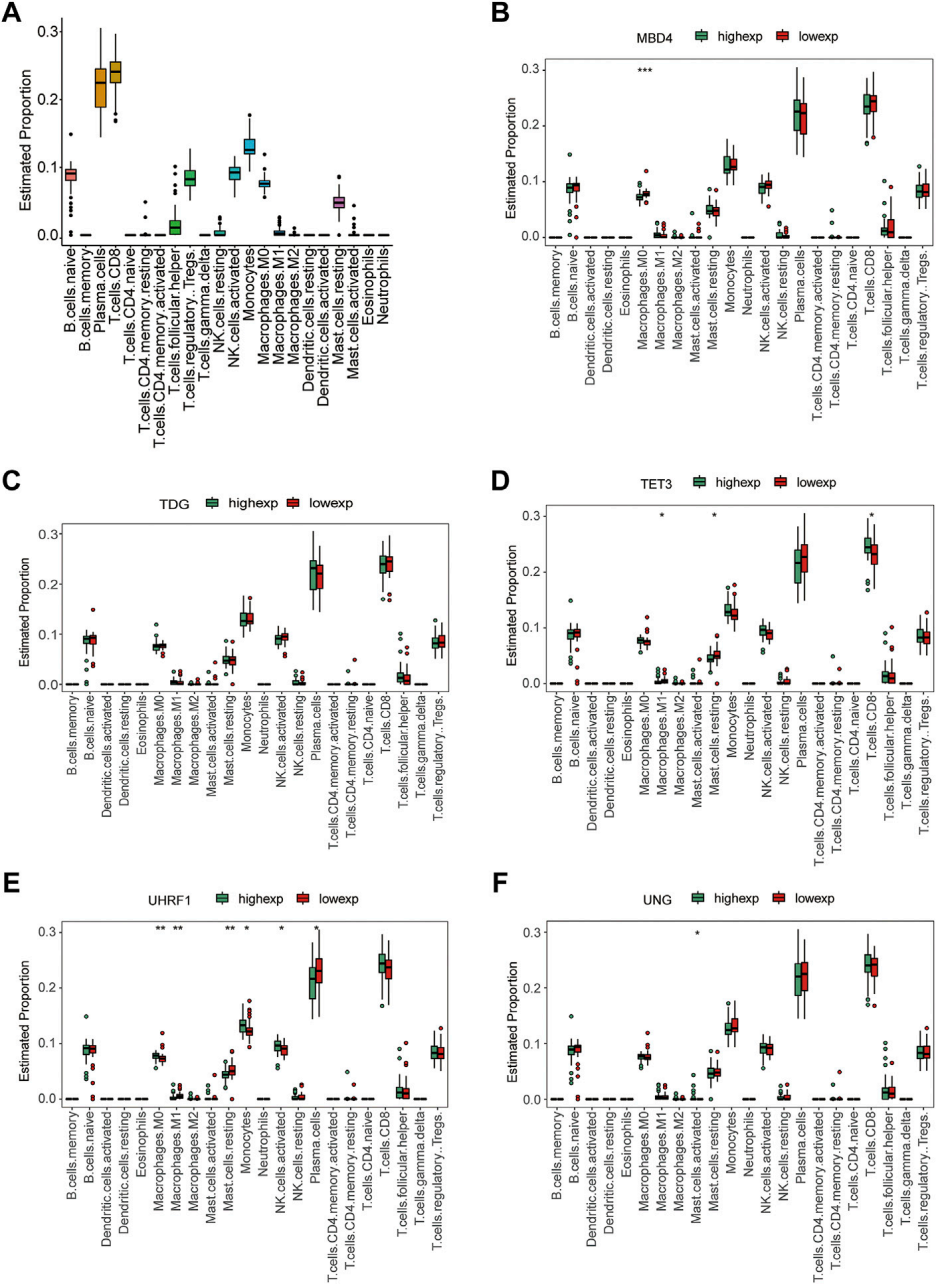
Immune infiltration analysis. **(A)** Boxplot of the proportion of 22 types of immune cells. **(B–F)** Immune cell infiltration characteristics between high gene expression and low gene expression groups: **(B)** *MBD4*, **(C)** *TDG*, **(D)** *TET3*, **(E)** *UHRF1*, and **(F)** *UNG*.

To identify the interactions between hub gene expression and immune infiltration, we first calculated the proportion of 22 immune cells in each sample using the CIBERSORT algorithm (Figure 11A). We also computed the correlation coefficients between the hub gene expression and immune cell infiltration levels. The results showed that the expression levels of the hub genes were significantly correlated with various immune cell proportions (Figures 11B–F). For example, the expression of *MBD4* was related to M0 macrophage proportion. The expression of *TET3* was correlated with the levels of M1 macrophages and CD8^+^ T cells. In addition, the expression levels of *UHRF1*, *UNG*, and other genes significantly correlated with various immune cells. These results indicate that the hub genes are related to the immune infiltration microenvironment from multiple perspectives.

## Discussion

T2D is a lifelong metabolic disorder with high prevalence worldwide. The exact pathogenesis of T2D remains unclear, and drug therapy for T2D involves lowering blood sugar levels (Draznin et al., 2022). Therefore, additional insights into T2D pathophysiology and treatment are urgently needed to improve the clinical management of T2D. Increasing evidence shows that post-transcriptional RNA modifications play an important role in diabetes (Jonkhout et al., 2017; Ling and Rönn, 2019). However, the role of m5C methylation as one of the most common RNA modifications in T2D remains unclear. Therefore, to investigate the functional role of m5C in T2D, we performed an integrative analysis of the merged gene expression profiles of 37 T2D patients and 36 controls. A total of 26 m5C-related genes and 58 T2D-related DEGs were identified. The expression of m5C-related genes was significantly correlated with the expression of most T2D-related DEGs. After LASSO regression, the *ZBTB4* gene was obtained, and we constructed a diagnostic model and validated its accuracy. GO, KEGG, and GSEA analyses indicated that these enriched pathways were closely related to metabolism-related biological processes, protein-containing complexes, cell death, and apoptotic processes in T2D. WGCNA showed that the turquoise module was associated with the most pyroptosis-related genes. Furthermore, the top five hub genes associated with T2D were screened from the PPI network based on the 12 m5C-related DEGs in the turquoise module. In addition, a ceRNA interaction network of hub m5C-related genes was obtained. Moreover, the expression levels of hub m5C-related genes were significantly correlated with the levels of various immune cells.

In the first part of this study, we identified 26 m5C-related genes in the merged dataset, which included 37 T2D and 36 normal skeletal muscle samples. The *TET* family members were highly expressed in the T2D group, whereas the *ZBTB* family members were highly expressed in the control group. Cluster analysis also supported this finding, showing that the expression levels of m5C-related genes could differentiate the two groups. This indicated that the m5C-related genes are associated with diabetes. We also identified 58 T2D-related genes from this dataset. The expression of m5C-related genes was significantly correlated with that of most T2D-related DEGs. For example, *DNMT1* expression was positively and negatively correlated with *NUCB1* and *MYBPC1* expressions, respectively. A significant correlation between the expression of m5C-related genes was also observed. m5C regulated epitranscriptome expression, mainly by modulating the binding of writer (methyltransferases), eraser (dimethyltransferases), and reader proteins (Trixl and Lusser, 2019). The *TET* family proteins function as RNA dimethylases, including *TET1*, *TET2*, and *TET3*, and their functions involve RNA degradation. A previous study has demonstrated that *TET2*, the most commonly regulated 5 mC dimethyl transferases, was characterized as demethylase in adipogenesis (Hou et al., 2020). Low expression levels of *TET2* have been correlated with poor prognosis in hepatocellular carcinoma (Jin et al., 2020). *ZBTB4*, also known as *KAISO-L1* or *ZNF903*, acts as a transcriptional repressor and is vital for maintaining mammalian genomic stability (Roussel-Gervais et al., 2017). Our study found that the *ZBTB* family was highly expressed in the control group, implying that altered *ZBTB* expression may play a role in T2D. Several studies have shown that *ZBTB4* is downregulated in multiple tumor types, such as breast cancer, Ewing sarcoma, and colorectal cancer (Kim et al., 2012; Yu et al., 2018; Xiang et al., 2020). Xiang et al. showed that high expression levels of *ZBTB4* were associated with a good prognosis in colorectal cancer (Xiang et al., 2020). Regarding the diagnostic value, we constructed a diagnostic model based on *ZBTB4*, which was obtained using LASSO regression analysis. The AUC value of the model was 0.655, suggesting that this model exhibited moderate accuracy (Akobeng, 2007) and may be an ideal target for the diagnosis of T2D. It has been documented that *ZBTB4* is involved in the regulation of diabetes-related complications (Zhu et al., 2019). *Zbtb7c* gene, which belongs to the ZBTB4 zinc finger protein family, has been identified as a critical gluconeogenic transcription factor (Choi et al., 2019).

In the second part of this study, we identified two molecular subtypes by performing unsupervised clustering for T2D samples based on 26 m5C-related genes. Six T2D-related DEGs (*IGFBP6*, *PDK4*, *RPS4Y1*, *S100A4*, *TPT1*, and *ZFP36*) significantly differed in their expression levels between the two molecular subtypes. *IGFBP6* and *PDK4* were upregulated in patients with diabetes and diabetes-related complications (Lu et al., 2012; Moon et al., 2012). A recent study found that the upregulated *RPS4Y1* in endothelial cells in a high-glucose environment may contribute to endothelial cell dysfunction by regulating the p38 MAPK signaling pathway (Chen et al., 2021). *S100A4* has been identified as a biomarker for insulin resistance (Anguita-Ruiz et al., 2020). *TPT1* regulates glucose metabolism and has been investigated as a drug target for type 2 diabetes (Jeon et al., 2021). Caracciolo et al. found that wild-type and *ZFP36* ^−/−^ mice became diabetic and obese under a high-fat diet and that *ZFP36* ^*−/−*^ mice exhibited improvements in insulin sensitivity (Caracciolo et al., 2018). These results suggested that these genes were also related to their typing, reflecting the validity and accuracy of the clustering results. We performed GO analysis to demonstrate the biological functions of DEGs. The top five most significant GO terms were cellular respiration, energy derivation by the oxidation of organic compounds, contractile fiber, sarcomere, and aerobic respiration. These results indicated multiple biological processes related to energy metabolism and further confirmed the accuracy of the identified gene set. A disturbance between cellular demands for energy and energy supply is a key element in the development of T2D (Steinberg, 2018). A recent study showed that abnormal cellular energy metabolism caused elevated blood glucose levels and was closely associated with insulin resistance (Dulkadiroğlu et al., 2021). For KEGG pathway analysis, 24 pathways were enriched, including oxidative phosphorylation, thermogenesis, propanoate metabolism, and carbon metabolism. These results can also complement the GO enrichment results. GSEA was used to identify the functional enrichment of the molecular subtypes, and six significant GO-term enrichments were identified. The most enriched terms were protein-containing complexes, cell death, and apoptosis process. Accumulating evidence has demonstrated that the pathogenesis of T2D involves multiple types of programmed cell death, including apoptosis, autophagy, pyroptosis, and ferroptosis (Demirtas et al., 2016; Mamun et al., 2021; Sha et al., 2021). GSVA was also performed between the two clusters, and 4,091 pathways with significant differences were identified. These included palmitoyltransferase activity, collecting duct development, and telencephalon regionalization. Previous studies have reported high levels of circulating fatty acids in prediabetes and T2D patients, and excessive tissue exposure to fatty acids can result in insulin resistance (Serlie et al., 2007). Carnitine palmitoyltransferase I is the rate-limiting enzyme in fatty acid oxidation, and increased carnitine palmitoyltransferase I activity can accelerate fatty acid oxidation (Qu et al., 2016). In summary, these results indicate that energy metabolism and programmed cell death may be the major contributors to T2D.

In the third part of the present study, we identified 18 m5C-related DEGs between these two molecular subtypes. Then, we found there were interactions between the differentially expressed m5C-related genes in patient typing. Gene-gene interactions have been recognized to be important for understanding genetic causes of type 2 diabetes (Zhou et al., 2018; Banerjee et al., 2019). Diabetes mellitus is an autoimmune disease characterized by chronic inflammation and metabolic disorders (de Candia et al., 2019). Pyroptosis, a type of programmed cell death related to the response to innate immunity, has received more attention in the onset and progression of diabetes and its complications (Mamun et al., 2021). Most studies on the identification of hub genes have compared gene expression profiles between the control and disease groups (Che et al., 2019; Lin et al., 2020; Zhu et al., 2020). However, the effects of m5C modification or its connections with pyroptosis genes in T2D have not yet been reported. To determine the relationship between diabetes and pyroptosis, we conducted a WGCNA analysis to identify the pyroptosis module. The turquoise module was associated with most pyroptosis-related genes.

In the PPI network constructed in this study, five differentially expressed m5C-related genes in the turquoise module were identified as hub genes. *MBD4*, *TDG*, *UHRF1*, *UNG*, and *TET3* are recognized as binding proteins in the dynamic regulation of m5C. *TET3* has been involved in T2D by inducing *HNF4α* fetal isoform expression (Li D. et al., 2020). The mRNA expression of *TET3* was upregulated in diabetic patients and rats. Upregulated *TET3* expression can affect the dynamic regulation of m5C in T2D (Yuan et al., 2019). The ceRNA regulatory role of long non-coding RNA (lncRNA) in T2D has been identified (Lin et al., 2017). Recent studies revealed that m5C-related lncRNA signatures play important role in human cancers (Wang et al., 2021; Zheng et al., 2022). In this study, we constructed a ceRNA interaction network. Seven lncRNAs, 11 miRNAs, and five mRNAs were identified, suggesting that post-transcriptional regulation played a role in the expression of key genes related to T2D.

Recently, immune system-based treatments for many diseases have emerged, including cancer and diabetes. In animal models, targeting immune cells enhances or suppresses diabetes development. There is increasing evidence that components of the innate immune system contribute significantly to T2D (Dalmas, 2019). Our current study showed that the expression of hub genes was significantly associated with the levels of various immune cells. For example, *MBD4* expression was related to M0 macrophage levels. *TET3* expression correlated with the levels of M1 macrophages and CD8^+^ T cells. The expression of *UHRF1*, *UNG,* and other genes was significantly correlated with the levels of various immune cells. Unlike CD4^+^ regulatory T cells, CD8^+^ T and CD4^+^ T helper one counterparts promoted insulin resistance (Shu et al., 2012). Jin et al. found that hyperglycemia induces M1 macrophage activation and increased the expression of inflammatory genes through the NF-κB pathway (Jin et al., 2015). Ahmed et al. reviewed epigenetic mechanisms, including DNA methylation and the regulation of macrophage activation in T2D (Ahmed et al., 2017). Together, these findings imply that these hub genes play a vital role in the recruitment and regulation of immune-infiltrating cells in T2D.

Our study had some limitations. First, to fully understand the role of m5C-related genes in T2D, a comprehensive evaluation of blood samples and skeletal muscle tissues is required. Second, our study validated the diagnostic accuracy of *ZBTB4* expression for T2D; further external validation employing a larger sample size will help authenticate the results. Third, the lack of detailed clinical data precluded an evaluation of the relationships between risk factors and molecular subtypes according to T2D complications. For instance, more severe inflammation appears in T2D patients with clinical complications. More detailed clinical features of T2D patients should be included in the future for further subset analysis. Finally, full clarification of the function of hub genes in T2D will require further experimental investigation, such as quantitative real-time PCR, western blot analysis, and immunohistochemical assays.

In conclusion, our results demonstrate that 5-methylcytosine methylation plays a role in T2D, broadening our knowledge of its pathophysiology. Furthermore, we believe this hypothesis-generating study provides new insights into the molecular mechanisms underlying T2D and identifies several potential biomarkers for its diagnosis and treatment.

## Data Availability

The datasets presented in this study can be found in online repositories. The names of the repository/repositories and accession number(s) can be found in the article/Supplementary Material.
