# Supplementary material for: Comprehensive analysis of key m5C modification-related genes in type 2 diabetes
Source: Front Genet. 2022 Oct 6;13:1015879. doi: 10.3389/fgene.2022.1015879 (PMC9582283; doi:10.3389/fgene.2022.1015879)
Supplement: Supplementary file 2 [file Presentation1.PDF]

Supplementary Material

Supplementary Figure 1. Landscape of m5C-related genes. (A) Expression profile without batch effect correction and standardized by Z-score. (B) Expression profile after batch effect correction and Z-score standardization.
