# Supplementary figures and images for: Comprehensive analysis of key m5C modification-related genes in type 2 diabetes
Source: Front Genet. 2022 Oct 6;13:1015879. doi: 10.3389/fgene.2022.1015879 (PMC9582283; doi:10.3389/fgene.2022.1015879)

A

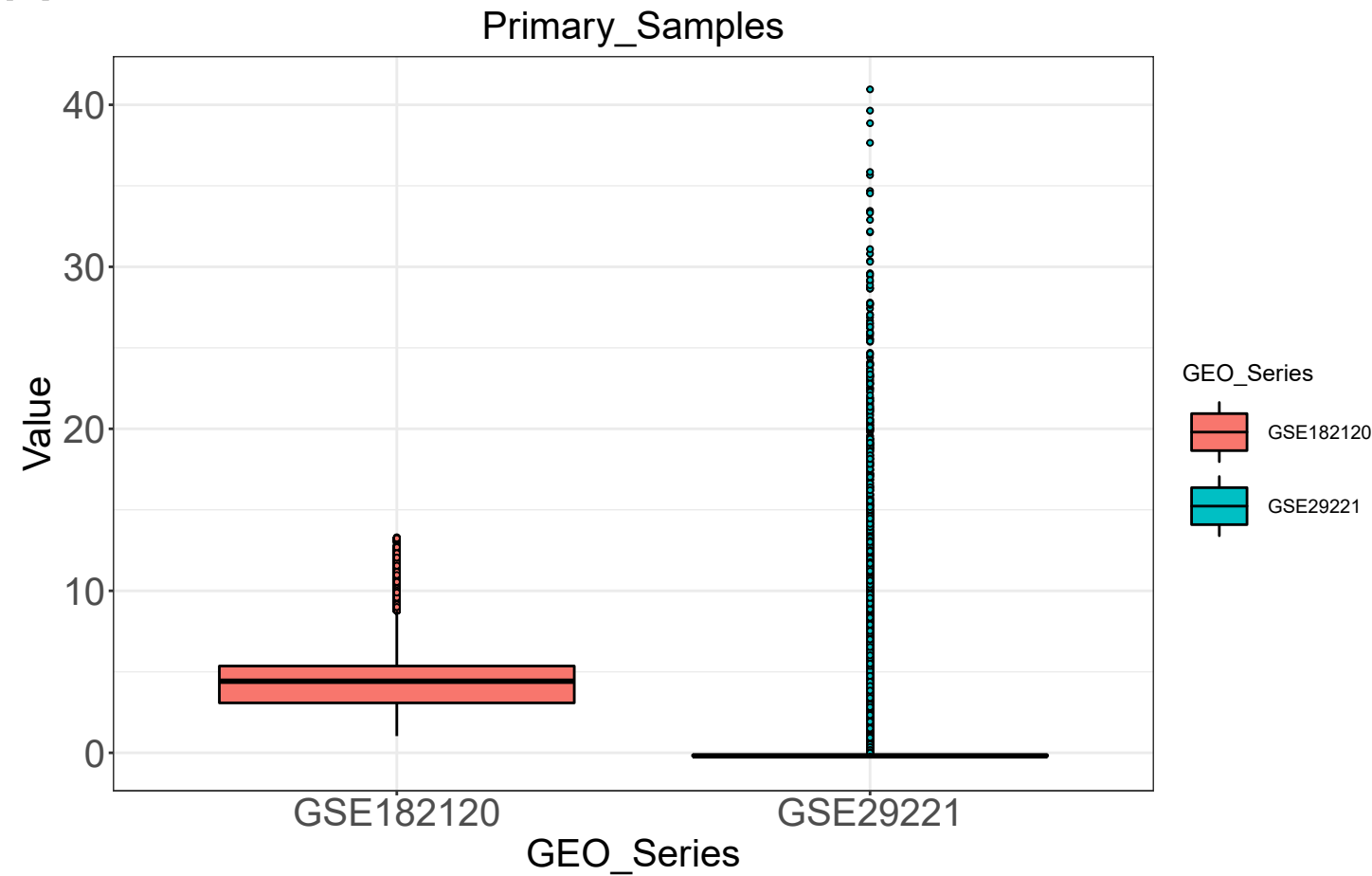

B

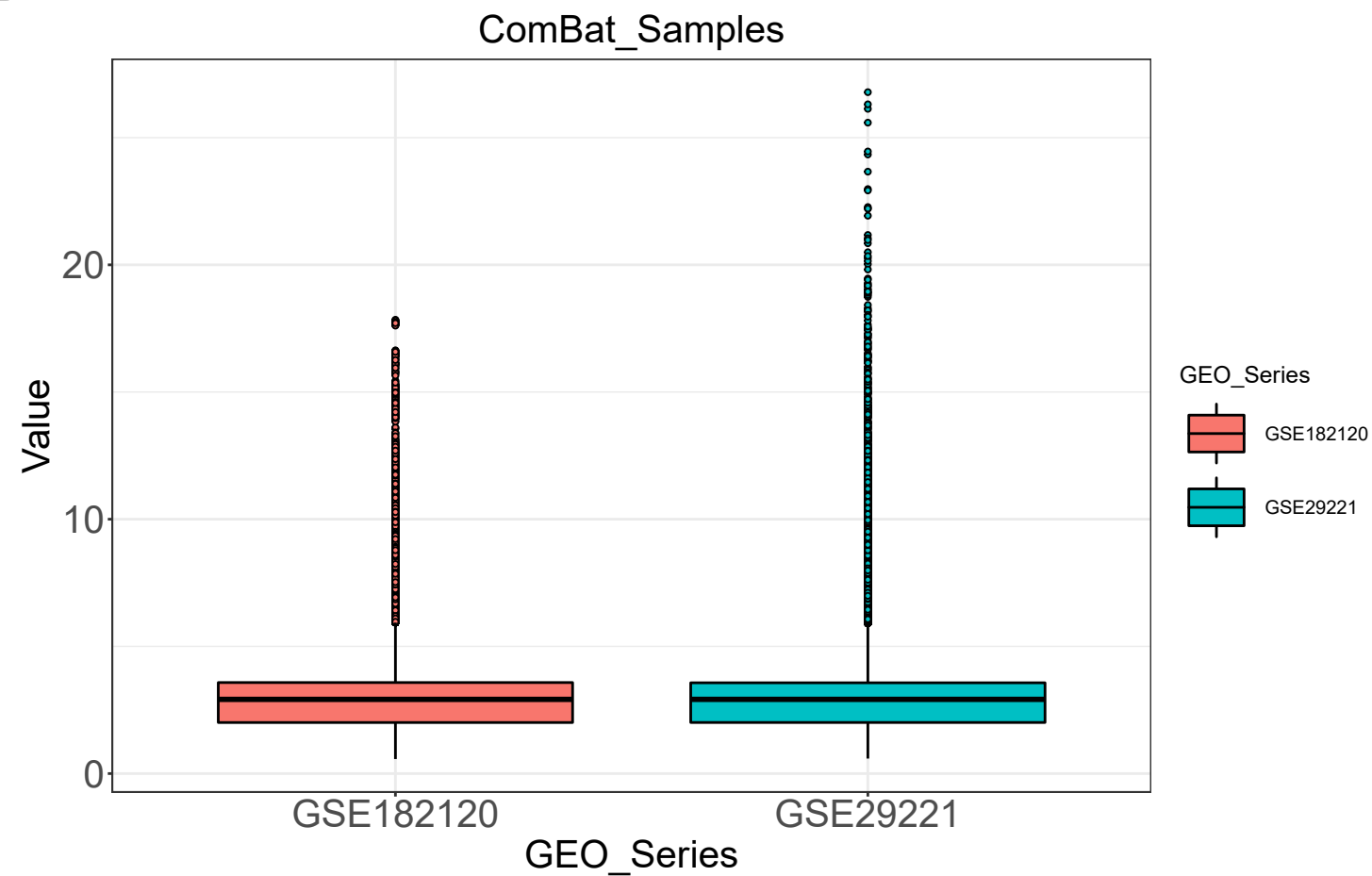

Supplement: Supplementary file 4 [file Image1.PDF]
